# 4-Meth­oxy-*N*-methyl­benzamide

**DOI:** 10.1107/S1600536812004746

**Published:** 2012-02-10

**Authors:** Juan Yuan, Yan-Ju Liu

**Affiliations:** a Pharmacy College, Henan University of Traditional Chinese Medicine, Zhengzhou 450008, People’s Republic of China

## Abstract

In the title compound, C_9_H_11_NO_2_, the dihedral angle between the amide group and the benzene ring is 10.6 (1)°. In the crystal, mol­ecules are connected *via* N—H⋯O hydrogen bonds, supported by a C—H⋯O contact, forming chains along *b*. These chains are linked by C—H⋯π inter­actions to give a three-dimensional network.

## Related literature

The title compound is an important inter­mediate in organic synthesis. For background to applications of the title compound and the synthesis, see: Lee *et al.* (2009 ▶). For bond-length data, see: Allen *et al.* (1987 ▶).
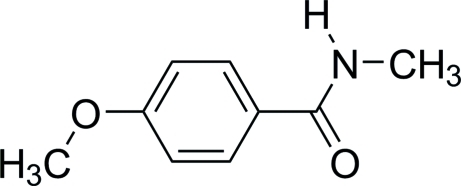


## Experimental

### 

#### Crystal data

C_9_H_11_NO_2_
*M* *_r_* = 165.19
Monoclinic, 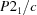

*a* = 8.7350 (17) Å
*b* = 9.2750 (19) Å
*c* = 10.719 (2) Å
β = 99.83 (3)°
*V* = 855.7 (3) Å^3^
*Z* = 4
Mo *K*α radiation
μ = 0.09 mm^−1^
*T* = 293 K
0.30 × 0.20 × 0.10 mm

#### Data collection

Enraf–Nonius CAD-4 diffractometer
Absorption correction: ψ scan (North *et al.*, 1968 ▶) *T*_min_ = 0.973, *T*_max_ = 0.991
3239 measured reflections
1573 independent reflections
1088 reflections with *I* > 2σ(*I*)
*R*_int_ = 0.042
3 standard reflections every 200 reflections intensity decay: 1%

#### Refinement

*R*[*F* ^2^ > 2σ(*F* ^2^)] = 0.052
*wR*(*F* ^2^) = 0.169
*S* = 1.00
1573 reflections
110 parameters
H-atom parameters constrained
Δρ_max_ = 0.18 e Å^−3^
Δρ_min_ = −0.19 e Å^−3^

### 

Data collection: *CAD-4 Software* (Enraf–Nonius, 1985 ▶); cell refinement: *CAD-4 Software*; data reduction: *XCAD4* (Harms & Wocadlo, 1995 ▶); program(s) used to solve structure: *SHELXS97* (Sheldrick, 2008 ▶); program(s) used to refine structure: *SHELXL97* (Sheldrick, 2008 ▶); molecular graphics: *SHELXTL* (Sheldrick, 2008 ▶); software used to prepare material for publication: *SHELXTL*.

## Supplementary Material

Crystal structure: contains datablock(s) I, global. DOI: 10.1107/S1600536812004746/sj5190sup1.cif

Structure factors: contains datablock(s) I. DOI: 10.1107/S1600536812004746/sj5190Isup2.hkl

Supplementary material file. DOI: 10.1107/S1600536812004746/sj5190Isup3.cml

Additional supplementary materials:  crystallographic information; 3D view; checkCIF report

## Figures and Tables

**Table 1:** Hydrogen-bond geometry (Å, °) *Cg*1 is the centroid of the C1–C6 benzene ring.

*D*—H⋯*A*	*D*—H	H⋯*A*	*D*⋯*A*	*D*—H⋯*A*
N—H0*A*⋯O2^i^	0.86	2.20	2.961 (2)	147
C1—H1*A*⋯O2^i^	0.93	2.46	3.378 (3)	169
C7—H7*C*⋯*Cg*1^ii^	0.96	2.94	3.816 (3)	153

Symmetry codes: (i) 
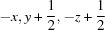
; (ii) 
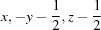
.

**Fig. 1.**
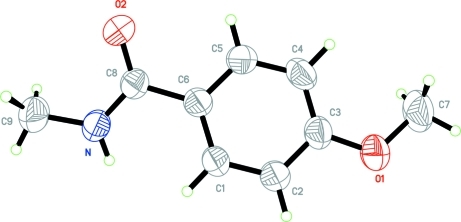
The molecular structure of (I), with the atom-numbering scheme. Displacement ellipsoids are drawn at the 50% probability level.

**Fig. 2.**
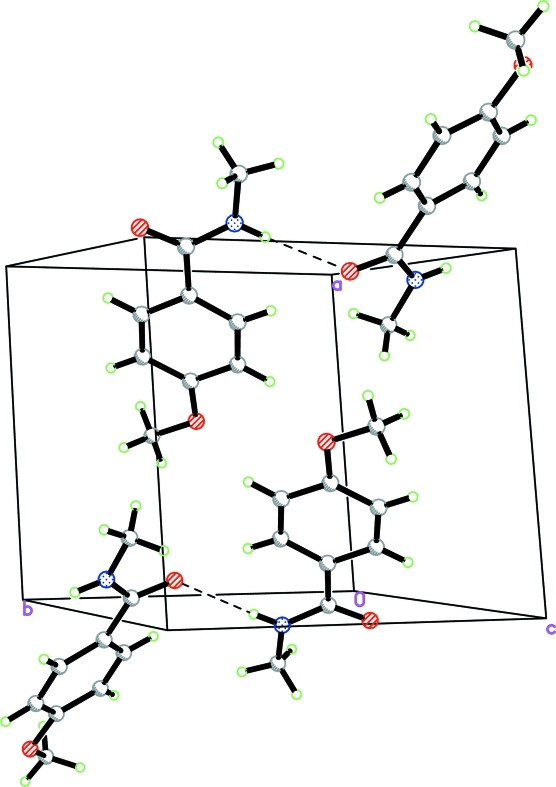
A packing diagram of (I).

## supplementary crystallographic information

### Comment

Benzamide derivatives exhibit interesting biological activities including antibacterial and antifungal effects (Lee *et al.*, 2009). We report here the crystal structure of the title compound 4-methoxy-*N*-methylbenzamide, (I).

The molecular structure of (I) is shown in Fig. 1. The dihedral angle between the amide group and the benzene ring is 10.6 (1)°. The bond lengths are within normal ranges (Allen *et al.*, 1987). In the crystal structure, intermolecular N—H0A···O2 hydrogen bonds, supported by C1—H1···O1 contacts (Table 1) result in the molecular chains along *b*. These chains are linked by C7—H7···π interactions to give a three-dimensional network.

### Experimental

The title compound, (I) was prepared by a literature method (Lee *et al.*, 2009). Crystals were obtained by dissolving (I) (0.2 g) in methanol (50 ml) and evaporating the solvent slowly at room temperature for about 10 d.

### Refinement

All H atoms were positioned geometrically and constrained to ride on their parent atoms, with C—H = 0.93 Å for aromatic H, 0.96 Å for methyl H and 0.86 Å for N—H, respectively. The *U*_iso_(H) = *xU*_eq_(C), where *x* = 1.2 for aromatic H and N—H, and *x* = 1.5 for methyl H.

### Crystal data

**Table:**

C_9_H_11_NO_2_	*F*(000) = 352
*M**_r_* = 165.19	*D*_x_ = 1.282 Mg m^−^^3^
Monoclinic, *P*2_1_/*c*	Mo *K*α radiation, λ = 0.71073 Å
Hall symbol: -P 2ybc	Cell parameters from 25 reflections
*a* = 8.7350 (17) Å	θ = 10–14°
*b* = 9.2750 (19) Å	µ = 0.09 mm^−^^1^
*c* = 10.719 (2) Å	*T* = 293 K
β = 99.83 (3)°	Block, colourless
*V* = 855.7 (3) Å^3^	0.30 × 0.20 × 0.10 mm
*Z* = 4	

### Data collection

**Table:**

Enraf–Nonius CAD-4 diffractometer	1088 reflections with *I* > 2σ(*I*)
Radiation source: fine-focus sealed tube	*R*_int_ = 0.042
Graphite monochromator	θ_max_ = 25.4°, θ_min_ = 2.4°
ω/2θ scans	*h* = 0→10
Absorption correction: ψ scan (North *et al.*, 1968)	*k* = −11→11
*T*_min_ = 0.973, *T*_max_ = 0.991	*l* = −12→12
3239 measured reflections	3 standard reflections every 200 reflections
1573 independent reflections	intensity decay: 1%

### Refinement

**Table:**

Refinement on *F*^2^	Secondary atom site location: difference Fourier map
Least-squares matrix: full	Hydrogen site location: inferred from neighbouring sites
*R*[*F*^2^ > 2σ(*F*^2^)] = 0.052	H-atom parameters constrained
*wR*(*F*^2^) = 0.169	*w* = 1/[σ^2^(*F*_o_^2^) + (0.1*P*)^2^ + 0.095*P*] where *P* = (*F*_o_^2^ + 2*F*_c_^2^)/3
*S* = 1.00	(Δ/σ)_max_ < 0.001
1573 reflections	Δρ_max_ = 0.18 e Å^−^^3^
110 parameters	Δρ_min_ = −0.19 e Å^−^^3^
0 restraints	Extinction correction: *SHELXL97* (Sheldrick, 2008), Fc^*^=kFc[1+0.001xFc^2^λ^3^/sin(2θ)]^-1/4^
Primary atom site location: structure-invariant direct methods	Extinction coefficient: 0.25 (2)

### Special details

**Table:**

Geometry. All e.s.d.'s (except the e.s.d. in the dihedral angle between two l.s. planes) are estimated using the full covariance matrix. The cell e.s.d.'s are taken into account individually in the estimation of e.s.d.'s in distances, angles and torsion angles; correlations between e.s.d.'s in cell parameters are only used when they are defined by crystal symmetry. An approximate (isotropic) treatment of cell e.s.d.'s is used for estimating e.s.d.'s involving l.s. planes.
Refinement. Refinement of *F*^2^ against ALL reflections. The weighted *R*-factor *wR* and goodness of fit *S* are based on *F*^2^, conventional *R*-factors *R* are based on *F*, with *F* set to zero for negative *F*^2^. The threshold expression of *F*^2^ > σ(*F*^2^) is used only for calculating *R*-factors(gt) *etc*. and is not relevant to the choice of reflections for refinement. *R*-factors based on *F*^2^ are statistically about twice as large as those based on *F*, and *R*-factors based on ALL data will be even larger.

### Fractional atomic coordinates and isotropic or equivalent isotropic displacement parameters (Å^2^)

**Table:**

	*x*	*y*	*z*	*U*_iso_*/*U*_eq_	
N	−0.0727 (2)	0.35158 (19)	0.24974 (17)	0.0581 (6)	
H0A	−0.0375	0.4382	0.2598	0.070*	
O1	0.4704 (2)	0.42231 (19)	0.72531 (15)	0.0746 (6)	
C1	0.1914 (3)	0.4288 (2)	0.4363 (2)	0.0641 (7)	
H1A	0.1610	0.4937	0.3706	0.077*	
O2	−0.0596 (2)	0.12586 (17)	0.32506 (15)	0.0701 (6)	
C2	0.3083 (3)	0.4659 (2)	0.5335 (2)	0.0701 (8)	
H2A	0.3569	0.5550	0.5321	0.084*	
C3	0.3548 (3)	0.3727 (2)	0.63338 (19)	0.0544 (6)	
C4	0.2849 (3)	0.2390 (3)	0.63200 (19)	0.0564 (6)	
H4A	0.3161	0.1740	0.6975	0.068*	
C5	0.1686 (3)	0.2022 (2)	0.5331 (2)	0.0532 (6)	
H5A	0.1229	0.1116	0.5328	0.064*	
C6	0.1180 (2)	0.2963 (2)	0.43448 (19)	0.0474 (6)	
C7	0.5133 (3)	0.3375 (3)	0.8361 (2)	0.0813 (9)	
H7A	0.5957	0.3847	0.8921	0.122*	
H7B	0.5481	0.2445	0.8131	0.122*	
H7C	0.4253	0.3260	0.8779	0.122*	
C8	−0.0109 (2)	0.2511 (2)	0.33215 (18)	0.0497 (6)	
C9	−0.1962 (3)	0.3222 (3)	0.1440 (2)	0.0700 (8)	
H9A	−0.2229	0.4094	0.0970	0.105*	
H9B	−0.2857	0.2867	0.1753	0.105*	
H9C	−0.1618	0.2512	0.0898	0.105*	

### Atomic displacement parameters (Å^2^)

**Table:**

	*U*^11^	*U*^22^	*U*^33^	*U*^12^	*U*^13^	*U*^23^
N	0.0653 (12)	0.0475 (10)	0.0548 (11)	−0.0002 (9)	−0.0089 (9)	−0.0047 (8)
O1	0.0820 (12)	0.0739 (11)	0.0562 (10)	−0.0104 (9)	−0.0210 (9)	0.0078 (8)
C1	0.0857 (17)	0.0445 (12)	0.0520 (13)	−0.0056 (12)	−0.0170 (12)	0.0065 (10)
O2	0.0913 (13)	0.0485 (9)	0.0628 (10)	−0.0150 (8)	−0.0085 (9)	−0.0023 (7)
C2	0.0898 (18)	0.0466 (12)	0.0629 (14)	−0.0129 (12)	−0.0185 (13)	0.0059 (10)
C3	0.0585 (13)	0.0559 (13)	0.0445 (11)	0.0014 (10)	−0.0034 (10)	−0.0015 (10)
C4	0.0685 (14)	0.0558 (13)	0.0429 (11)	0.0057 (11)	0.0045 (10)	0.0115 (10)
C5	0.0644 (14)	0.0459 (11)	0.0483 (12)	−0.0041 (10)	0.0065 (10)	0.0011 (9)
C6	0.0575 (12)	0.0411 (11)	0.0427 (11)	0.0032 (9)	0.0058 (9)	−0.0017 (8)
C7	0.0776 (17)	0.106 (2)	0.0522 (14)	−0.0006 (16)	−0.0108 (13)	0.0132 (14)
C8	0.0593 (13)	0.0460 (12)	0.0427 (11)	0.0008 (10)	0.0058 (9)	−0.0059 (9)
C9	0.0688 (15)	0.0703 (16)	0.0630 (16)	−0.0017 (12)	−0.0117 (13)	−0.0018 (12)

### Geometric parameters (Å, º)

**Table:**

N—C8	1.333 (3)	C4—C5	1.380 (3)
N—C9	1.451 (3)	C4—H4A	0.9300
N—H0A	0.8600	C5—C6	1.384 (3)
O1—C3	1.365 (3)	C5—H5A	0.9300
O1—C7	1.420 (3)	C6—C8	1.492 (3)
C1—C2	1.372 (3)	C7—H7A	0.9600
C1—C6	1.385 (3)	C7—H7B	0.9600
C1—H1A	0.9300	C7—H7C	0.9600
O2—C8	1.235 (3)	C9—H9A	0.9600
C2—C3	1.383 (3)	C9—H9B	0.9600
C2—H2A	0.9300	C9—H9C	0.9600
C3—C4	1.381 (3)		
			
C8—N—C9	123.30 (19)	C5—C6—C1	117.52 (19)
C8—N—H0A	118.3	C5—C6—C8	119.12 (19)
C9—N—H0A	118.3	C1—C6—C8	123.36 (19)
C3—O1—C7	118.3 (2)	O1—C7—H7A	109.5
C2—C1—C6	121.1 (2)	O1—C7—H7B	109.5
C2—C1—H1A	119.5	H7A—C7—H7B	109.5
C6—C1—H1A	119.5	O1—C7—H7C	109.5
C1—C2—C3	120.9 (2)	H7A—C7—H7C	109.5
C1—C2—H2A	119.6	H7B—C7—H7C	109.5
C3—C2—H2A	119.6	O2—C8—N	121.38 (19)
O1—C3—C4	125.57 (19)	O2—C8—C6	121.26 (19)
O1—C3—C2	115.6 (2)	N—C8—C6	117.36 (18)
C4—C3—C2	118.86 (19)	N—C9—H9A	109.5
C5—C4—C3	119.8 (2)	N—C9—H9B	109.5
C5—C4—H4A	120.1	H9A—C9—H9B	109.5
C3—C4—H4A	120.1	N—C9—H9C	109.5
C4—C5—C6	121.9 (2)	H9A—C9—H9C	109.5
C4—C5—H5A	119.1	H9B—C9—H9C	109.5
C6—C5—H5A	119.1		
			
C6—C1—C2—C3	−0.9 (4)	C4—C5—C6—C8	−178.5 (2)
C7—O1—C3—C4	−6.9 (4)	C2—C1—C6—C5	−1.0 (4)
C7—O1—C3—C2	174.2 (2)	C2—C1—C6—C8	179.2 (2)
C1—C2—C3—O1	−179.0 (2)	C9—N—C8—O2	−2.2 (3)
C1—C2—C3—C4	2.0 (4)	C9—N—C8—C6	178.6 (2)
O1—C3—C4—C5	179.8 (2)	C5—C6—C8—O2	−9.3 (3)
C2—C3—C4—C5	−1.4 (3)	C1—C6—C8—O2	170.5 (2)
C3—C4—C5—C6	−0.5 (3)	C5—C6—C8—N	169.8 (2)
C4—C5—C6—C1	1.7 (3)	C1—C6—C8—N	−10.4 (3)

### Hydrogen-bond geometry (Å, º)

Cg1 is the centroid of the C1–C6 benzene ring.

**Table:**

*D*—H···*A*	*D*—H	H···*A*	*D*···*A*	*D*—H···*A*
N—H0*A*···O2^i^	0.86	2.20	2.961 (2)	147
C1—H1*A*···O2^i^	0.93	2.46	3.378 (3)	169
C7—H7*C*···*Cg*1^ii^	0.96	2.94	3.816 (3)	153

Symmetry codes: (i) −*x*, *y*+1/2, −*z*+1/2; (ii) *x*, −*y*−1/2, *z*−1/2.
